# Maternal asthma activity and offspring asthma: a linked-data population study in Australia and Sweden

**DOI:** 10.1136/bmjresp-2025-004010

**Published:** 2026-06-28

**Authors:** Bronwyn K Brew, Vanessa E Murphy, Cecilia Lundholm, Helga Zoega, Alys Havard, Annelies L Robijn, Tong Gong, Awad I Smew, Gustaf Rejnö, Georgina Chambers, Catarina Almqvist

**Affiliations:** 1 Department of Medical Epidemiology and Biostatistics, Karolinska Institutet, Stockholm, Stockholm County, Sweden; 2 National Perinatal Epidemiology and Statistical Unit, Centre for Big Data Research in Health, University of New South Wales, Sydney, New South Wales, Australia; 3 School of Medicine and Public Health, The University of Newcastle Australia, Newcastle, New South Wales, Australia; 4 Centre of Public Health Sciences, University of Iceland, Reykjavík, Iceland; 5 School of Population Health, University of New South Wales, Sydney, New South Wales, Australia; 6 National Drug and Alcohol Research Centre, University of New South Wales, Sydney, New South Wales, Australia; 7 Department of Perioperative Medicine and Intensive Care, Karolinska University Hospital, Stockholm, Sweden; 8 Obstetrics and Gynecology Unit, Stockholm South General Hospital, Stockholm, Sweden; 9 Pediatric Allergy and Pulmonology Unit at Astrid Lindgren Children’s Hospital, Karolinska University Hospital, Stockholm, Sweden

**Keywords:** Asthma Epidemiology, Paediatric asthma, Asthma

## Abstract

**Background:**

Inhaled corticosteroid (ICS) use in pregnancy has shown associations with decreased offspring asthma in childhood, whereas exacerbations in pregnancy may increase the risk. However, these findings have not been tested in population-based cohorts with rigorous assessment of confounding structures. The aim was to describe asthma exacerbations and ICS utilisation during pregnancy and to assess causal pathways between pregnancy exacerbations, ICS use and offspring asthma.

**Methods:**

Register-based cohort study of pregnancies in New South Wales (NSW), Australia and Sweden. Maternal monthly asthma exacerbations (hospital visits and/or oral corticosteroid (OCS) dispensings) and ICS dispensing rates were calculated 12 months prepregnancy to 12 months post partum. Among mothers with asthma (35 194 NSW, 102 248 Sweden), Hazard Ratios (HR)s were calculated for pregnancy exacerbations or ICS use and offspring asthma adjusting for confounders including asthma severity. Associations between paternal exposures and offspring asthma and sibling analyses assessed the likelihood of unmeasured confounding.

**Results:**

Maternal asthma prevalence was 8.6% in NSW and 7.1% in Sweden. While ICS use was maintained over the course of pregnancy, a postpartum decline was observed, accompanied by a rise in OCS use. Adjusted HRs for offspring asthma were 1.47 (95% CI 1.32 to 1.64, NSW) and 1.30 (95% CI 1.24 to 1.36, Sweden) following maternal exacerbations, and 1.37 (95% CI 1.26 to 1.49, NSW) and 1.23 (95% CI 1.19 to 1.27, Sweden) following maternal ICS use. Paternal exposures were also associated with offspring asthma, while associations in sibling analyses were not significant.

**Conclusions:**

Observed associations between maternal exacerbations, ICS use and offspring asthma appear to be due to unmeasured confounding. Management of maternal asthma remains a priority in pregnancy and post partum.

**WHAT IS ALREADY KNOWN ON THIS TOPIC**

- Asthma exacerbations in pregnancy have been shown to increase the risk of child asthma, while prenatal use of inhaled corticosteroids has been shown to decrease child asthma risk.

**WHAT THIS STUDY ADDS**

- In two separate large populations, maternal exacerbations and inhaled corticosteroid use in pregnancy were associated with increased asthma risk. However, we show evidence that these associations may be confounded by familial factors.

**HOW THIS STUDY MIGHT AFFECT RESEARCH, PRACTICE OR POLICY**

- Maternal ICS use and prevention of maternal exacerbations should remain a clinical priority despite not reducing offspring asthma risk. Other mechanisms for reducing offspring asthma should be explored.

## Introduction

 Approximately 1 in 10 pregnant women have asthma, with 33%–40% experiencing worsening symptoms during pregnancy (such as night-time symptoms, daily activity limitation) and 4%–10% experiencing severe asthma exacerbations.^1^,^7^ These exacerbations are associated with adverse perinatal outcomes including preterm birth and low birth weight.^8^,^12^ Despite the recognition of worsening asthma symptoms and potential consequences, the timing and specific patterns of exacerbations over the gestational period as hormonal and physiological changes are occurring are not well understood.

Inhaled corticosteroids (ICS) are recommended for asthma management during pregnancy and are considered safe for the fetus.^13^ Despite this recommendation, studies suggest that women decrease ICS use during early pregnancy, likely due to concerns about medication safety.^14^,^17^ This reduction can lead to more frequent exacerbations.^8^ Given recent changes in recommendations for all people with asthma including pregnant women to be taking ICSs^18^ it may be that ICS use in pregnancy has improved in recent years. Current data is needed describing the course of ICS use over pregnancy in relation to pre and postpartum rates and exacerbations in order to optimise maternal asthma care.

Furthermore, exacerbations and ICS use during pregnancy may have implications for offspring asthma risk. Although several studies have shown that exacerbations during pregnancy may increase the risk of offspring developing asthma compared with mild, controlled or no asthma,^3 19 20^ a recent meta-analysis failed to establish a clear link.^21^ Conversely, a clinical trial for guided asthma management during pregnancy found that ICS use during pregnancy reduced both maternal exacerbations and the risk of offspring asthma.^22^ Clarifying these relationships between asthma activity in pregnancy and offspring asthma is important for understanding the mechanisms of offspring asthma development, and for identifying preventive measures to reduce childhood asthma rates. Observational maternal–child studies may be vulnerable to unmeasured genetic or environmental confounding, such as lifestyle choices and proximity to traffic pollution. One method to assess unmeasured confounding is to use family designs such as paternal exposure during pregnancy (also known as a negative control) and sibling control analyses comparing exposed and non-exposed siblings.^23 24^ Such designs require very large populations with availability of family connections to fathers and sibling data which can be found in health administrative datasets.

This study leverages population-based linked data from two countries with a high prevalence of asthma. The objectives were to first describe maternal asthma exacerbations and ICS utilisation before, during and after pregnancy in two large populations of pregnant women, and second, to assess causal pathways between maternal asthma exacerbations and ICS utilisation with offspring asthma.

## Methods

### Data sources

From New South Wales (NSW), a population data linkage resource was used which includes all births of at least 22 weeks gestation and >400 g in NSW between 2001 and 2019.^25^ The NSW data resource links person-level perinatal data to health records (emergency department, hospital and pharmaceutical information) and demographic data for mothers, children and other parents. Similarly in Sweden, the data resource was a person-level national perinatal and health data linkage of births 1973 to 2022 linked to parental information.^26^,^28^ Patients or the public were not involved in the design, or conduct, or reporting, or dissemination plans of our research.

### Study populations

For the descriptive analyses before, during and after pregnancy, all women who gave birth in NSW between October 2014 and December 2018^25^ (n=407 879), and in Sweden between July 2008 and June 2021 (n=1 443 139) were included. These study populations are referred to as ‘population cohorts’.

For the causal analyses assessing maternal asthma in pregnancy and offspring asthma, the study populations were restricted to pregnant women with asthma and their offspring, identified from the population cohorts (NSW n=35 194, Sweden n=102 248). These are referred to as ‘maternal asthma cohorts’. Asthma eligibility was set at conception (last menstrual period) to ensure eligibility preceded exposure to minimise selection bias. Eligibility was defined as: an asthma medication dispensed or a hospitalisation, emergency department or outpatient visit (Sweden only) for asthma in the 18 months prior to conception. See online supplemental methods for more information on data sources and Anatomical Therapeutic Classification (ATC) codes. Similar asthma cohorts were created for fathers (‘paternal asthma cohorts’).

### Exposures

Maternal asthma exacerbations were defined as one of the following types: a hospitalisation or emergency department visit for asthma AND/OR an oral corticosteroid (OCS) dispensing (prednisolone-ATC code H02AB06, prednisone-ATC code H02AB07) AND/OR ≥5 short-acting beta-agonists (SABA) dispensings between date of conception and birth (causal analysis only). ICS use was defined as dispensing of an ICS medication (glucocorticoids-ATC code R03BA, adrenergics in combination with corticosteroids–R03AK).

### Outcome

The outcome for this study was asthma in children, defined using a validated algorithm based on asthma medication and diagnosis in both populations.^29^ In children under 4.5 years, a primary diagnosis for asthma in the hospital (including outpatient in Sweden) or emergency department records was required and one of the following medication criteria: (1) ≥2 dispensings of ICS (alone or combined with LABA) and/or leukotriene receptor agonist and (2) ≥3 dispensings of SABA within 12 months of each other. In children over 4.5 years, either a diagnosis or medication dispensing criteria was sufficient. A cut-off of 4.5 years is used as per the validated algorithm^29^ so that the younger children whose asthma is more likely to be misclassified are given a more stringent criteria, thus reducing the risk of false positives.

### Identification and definition of confounders

Maternal and perinatal factors (age, smoking in pregnancy, maternal body mass index (BMI), maternal country of birth (ethnicity in directed acyclic graph (DAG)), season of birth, sociodemographic data (socioeconomic status, SES)) were identified in the perinatal data collections. Smoking in pregnancy and BMI were both reported at the first perinatal clinical visit (generally 11–15 weeks). BMI was categorised into underweight (<18.5 kg/m^2^), recommended weight (18.5 to <25 kg/m^2^), overweight (25 to <30 kg/m^2^) and obesity (≥30 kg/m^2^). Maternal country of birth was defined first as the ‘Nation of study site’, that is, Australia for NSW data and Sweden for the Swedish data. The remaining women were then categorised into Asia & Middle East, Europe (not including Sweden) and Other. In the NSW data, mother’s area of residence was mapped to Index of Relative Socio-economic Disadvantage scores, which are split into quintiles from most disadvantaged to least disadvantaged.^30^ In Sweden, the mother’s highest educational attainment at the time of the child’s birth was identified from the Longitudinal Integrated database for Health Insurance and Labour Market Studies. Asthma severity was based on medication dispensings in the 18 months prior to pregnancy according to the Global Initiative for Asthma 2014 guidelines relevant to our study period: no medication, SABA only, ICS monotherapy or ICS combinations (with LABA or LTRA).^9 31^

### Statistical analysis

First, a descriptive analysis of the NSW and Sweden population cohorts assessed the rate of asthma exacerbations and types of exacerbation per month per 100 000 pregnancies in the 12 months prepregnancy (to establish a baseline), during pregnancy, and then 12 months post partum. The numerators per month were: (1) the number of pregnancies experiencing at least one exacerbation of any type (hospitalisation, emergency room visit, OCS dispense), (2) the number of pregnancies with at least one OCS dispensing and (3) the number of pregnancies with at least one hospital/emergency visit. ICS dispensing rates per month per 100 pregnancies were calculated for the population cohorts and maternal and paternal asthma cohorts. The numerator per month was the total number of ICS dispensings in the cohort (a person could contribute multiple dispensings in 1 month).

Second, Cox proportional hazards regression was used to assess the associations between asthma exacerbations (any type) or ICS use during pregnancy and offspring asthma in the maternal asthma cohorts. The underlying time scale was child age. Follow-up started at birth and ended with the first of incident asthma (date of first medication dispensing or diagnosis in children meeting the outcome criteria), emigration date (Sweden only), death, end of the study period (31 December 2022). The sandwich estimator of variance was used to account for correlation caused by clustering of observations within families (siblings and/or multiple pregnancies). The proportional hazards assumption was tested based on Schoenfeld residuals. Confounders were determined by applying subject-specific knowledge to DAGs (see online supplemental methods and online supplemental figures S1 and S2) for definitions and identification of confounders.^32^ Asthma exacerbation analyses were adjusted for maternal BMI, SES, maternal country of birth, season of birth, smoking in pregnancy and additionally for asthma severity. Genetic propensity for asthma was also identified as a potential confounder but as it was unmeasured we could not include it. ICS use analyses were adjusted for BMI, SES, season of birth and additionally for maternal asthma exacerbation and severity. Sex of the child was included as an interaction term. Birth weight and other perinatal factors were not included as they are mediators on the causal pathway. In order to assess whether there was a linear association between multiple maternal exacerbations or ICS dispensings with child asthma, Cox proportional hazards regression models were used modelling the exposure in incremental steps of increasing number of exacerbations or ICS dispensings over pregnancy.

#### Paternal control analyses

Using the paternal asthma cohort as a negative control exposure (asthma exacerbation or ICS use by the father during the period of the mother’s pregnancy) assessed the likelihood of familial (genetic or shared environmental) confounding.^23^ A negative control is not expected to be causally linked with the outcome (child asthma). An observed association between the negative control and the outcome is therefore due to residual/unmeasured familial confounding (eg, genetic heritability of asthma or unmeasured environmental triggers such as lifestyle factors on both parent and child asthma). An assumption is made that the unmeasured confounding that is causing the unexpected association between paternal exposure and child outcome is similarly affecting the association between maternal exposure and child outcome based on assumptions of the child having shared genetics and shared early life with each parent. If no association is seen for the negative control exposure, then the assumption is made that there is no unmeasured confounding of the maternal association, and any observed association can be interpreted as a causal association between maternal exposure and child outcome.

#### Sibling analyses

Comparing siblings discordant for prenatal exposure (either asthma exacerbation or ICS use) controlled for familial (measured or unmeasured) confounding.^24^ Siblings were identified from within the cohorts as children with the same mother. A sibling analysis assumes that siblings are genetically related (50%) and grew up in the same environment (climate, parenting, pollution, pets, food types, etc). Therefore, if a comparison of exposed children with their unexposed siblings yields a non-null risk for the outcomes, this provides support for a causal relationship. No association would suggest that there is in fact no causal relationship and the observed association in the whole population study is due to familial confounding.

#### Sensitivity analyses

(1) Extension of ICS exposure window in pregnancy to 30 days before conception to include women who may be using an earlier dispensing of ICS into pregnancy; (2) Confounding by indication was addressed by first restricting the cohorts to women who had ≥2 ICS dispensings in the 27 months prior to pregnancy so we could capture asthma severity more accurately using ICS dosage. Average number of defined daily doses (DDD) per day was calculated as dispensed DDDs divided by number of days between dispensings and (3) Subgroup analysis (with and without reported BMI data) of NSW maternal exposures unadjusted and adjusted without BMI to assess if the large amount of missing BMI data (31.7%) may potentially be causing bias.

All analyses were performed using SAS software V.9.4 (SAS Institute). Authors had direct access to the linked databases provided from the administrative authorities in each country which was kept in a secure repository in each country. Data cleaning was done by the authors.

## Results

### Descriptive analyses of maternal asthma exacerbations and ICS use

The prevalence of asthma in the maternal population cohorts was 8.6% in NSW and 7.1% in Sweden. Both populations had an average maternal age of 30 years, with NSW women having a higher rate of obesity (18% vs 13.9%) and smoking (6.4% vs 5.2%) (online supplemental Table S1). Maternal asthma cohorts revealed similar differences in population characteristics although with higher rates than general populations, for example, obesity: 28.4% NSW, 19.8% Sweden and smoking rates: 12.8% NSW, 7.2% Sweden (online supplemental Table S2).

**Figure 1:**
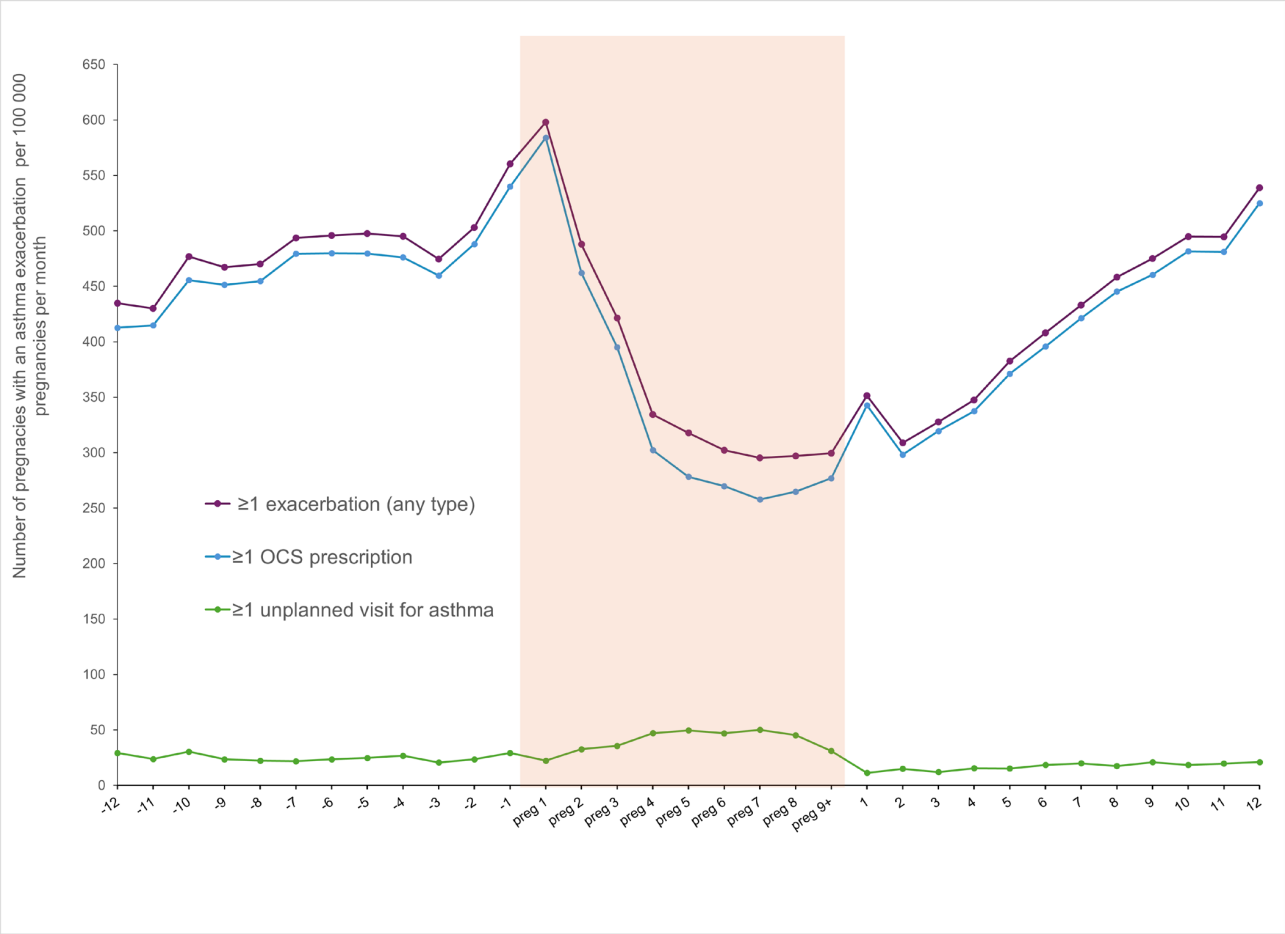
Asthma exacerbations in women giving birth in NSW, Australia October 2014–December 2018. Number of pregnancies with at least one exacerbation of any type (purple) per 100 000 pregnancies per month, broken down by oral corticosteroids (OCS) (blue) and unplanned visits to hospital or emergency department (green). Measured from 12 months prior to pregnancy, during pregnancy (pink box) to 12 months after pregnancy. NSW, New South Wales.

Asthma exacerbations were more prevalent at all time points before, during and post partum in the NSW population cohort compared with the Swedish cohort (figures1  2). In both populations, the most common exacerbation type was use of OCS, whereas unplanned visits to the emergency department or hospitalisation contributed less than 10% of data. Both populations experienced stable exacerbation rates prepregnancy and a peak in the first month of pregnancy, particularly pronounced in NSW (figure 1). During pregnancy, there was a rapid decline in exacerbations as identified by lower OCS dispensing, while unplanned medical visits slightly increased. After childbirth, OCS dispensing and asthma exacerbation rates rose over the next 11 months, eventually returning to prepregnancy rates.

**Figure 2:**
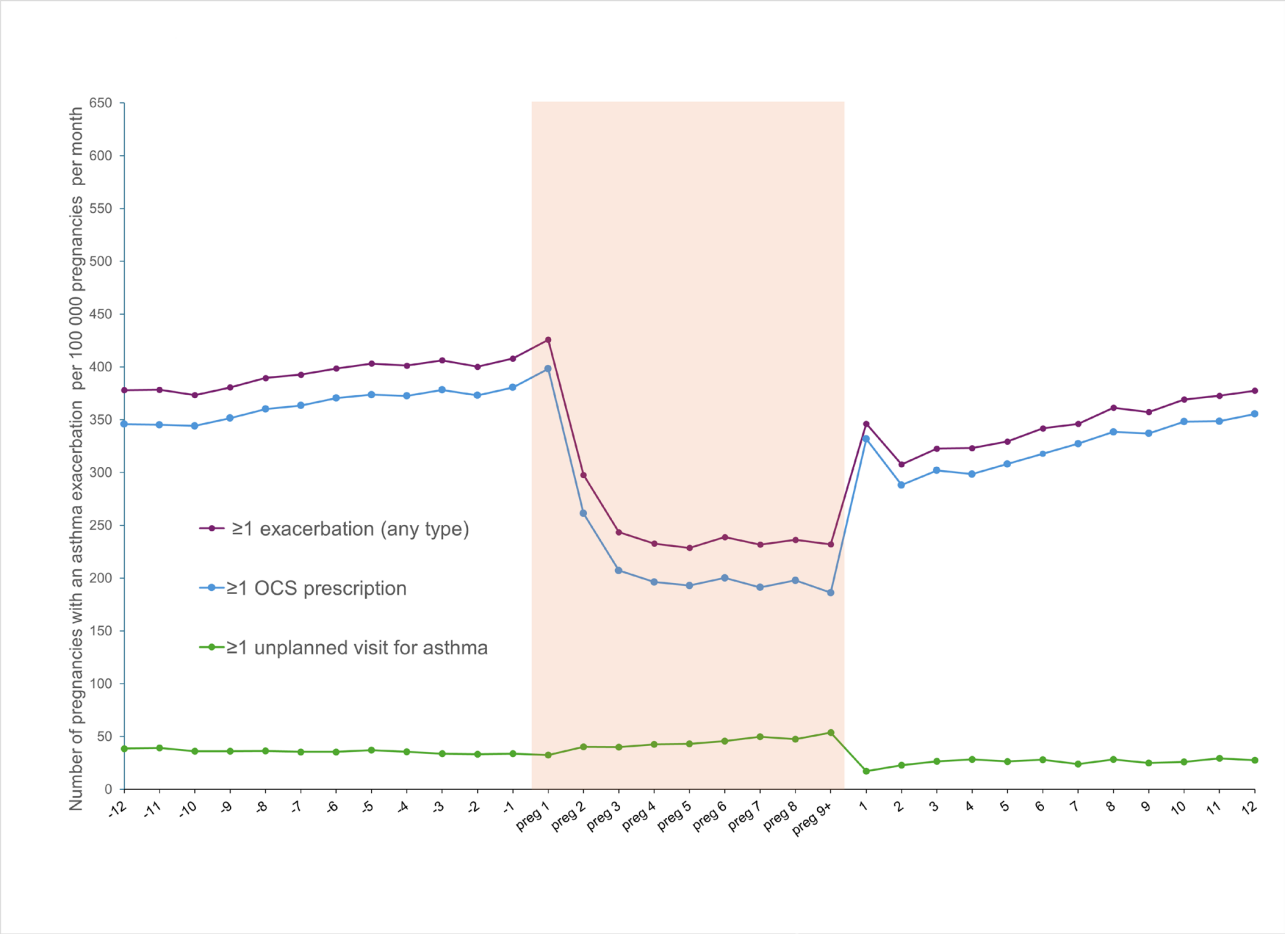
Asthma exacerbations in women giving birth in Sweden July 2008–June 2021. Number of pregnancies with at least one exacerbation of any type (purple) per 100 000 pregnancies per month, broken down by oral corticosteroids (OCS) (blue) and unplanned visits to hospital or emergency department (green). Measured from 12 months prior to pregnancy, during pregnancy (pink box) to 12 months after pregnancy.

**Figure 3:**
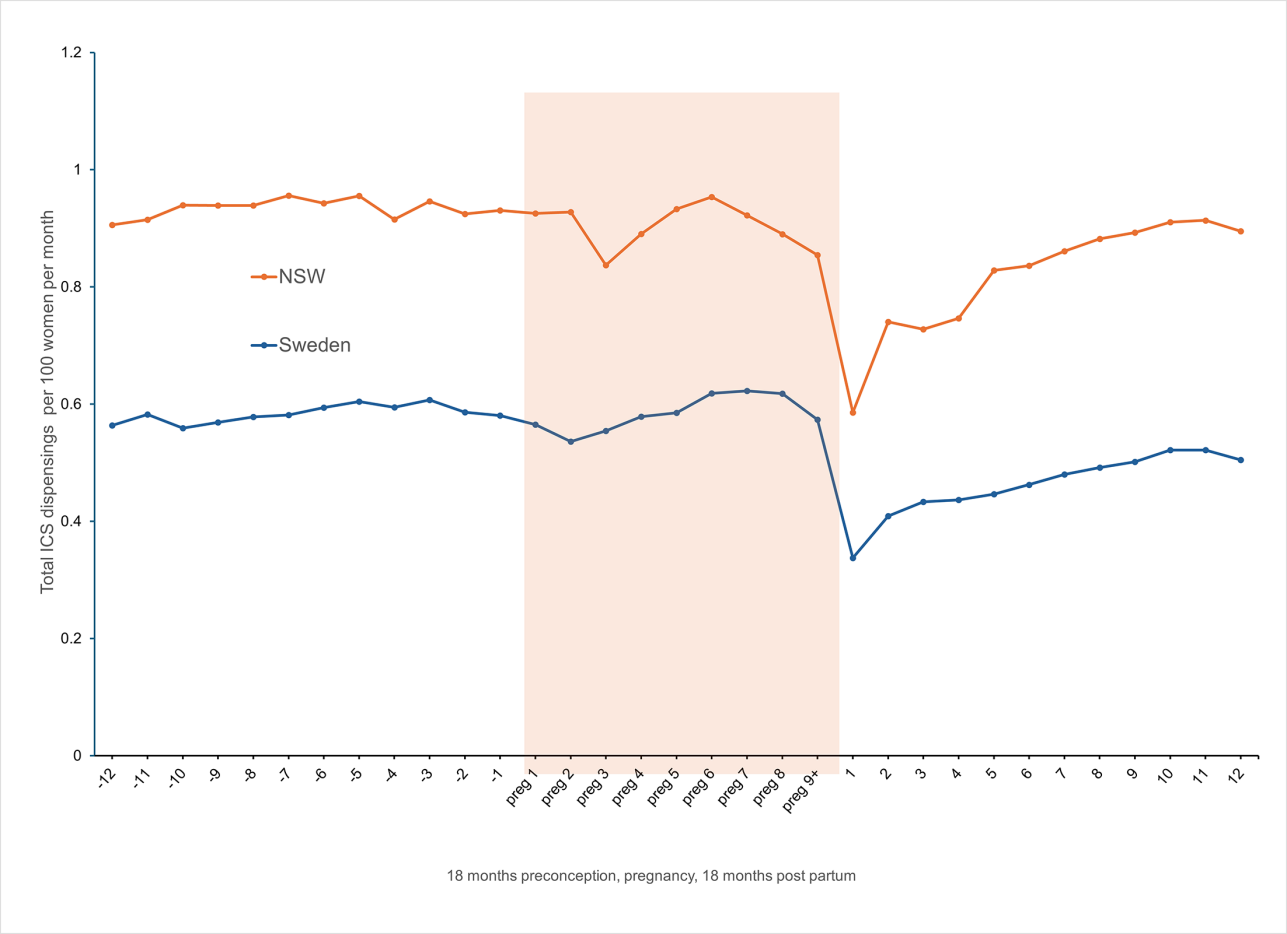
Rate of total ICS dispensings per 100 women per month in NSW, Australia (births October 2014–December 2018) and Sweden (births July 2008–June 2021). Measured from 12 months prior to pregnancy, during pregnancy (pink box) to 12 months after pregnancy. ICS, Inhaled corticosteroid; NSW, New South Wales.

ICS use was more prevalent at all time points in the NSW population cohort compared with the Swedish cohort (figure 3). Both population cohorts showed a drop in ICS use during the second (Sweden) and third (NSW) months of pregnancy, which returned to prepregnancy use before birth, with a slight decline in the 9th month and a large decline immediately after childbirth. ICS use returned to prepregnancy rates in NSW while Sweden remained low by 12 months post partum (figure 3). Similar ICS use patterns were also observed for both maternal asthma cohorts, whereas the paternal asthma cohorts had a steady ICS dispensing rate except for a spike in the last month of pregnancy (online supplemental figure S3).

### Causal assessment of prenatal exacerbations and ICS use on child asthma

In NSW, follow-up time ranged between 0 and 8 years and in Sweden from 0 to 15 years. Median age for asthma onset in offspring was 5.7 years in NSW and 5.1 years in Sweden, with asthma incidence rates of 28.1 and 40.5 episodes per 1000 person years in NSW and Sweden, respectively.

During pregnancy, the maternal asthma cohorts experienced exacerbation rates of 9.6% (n=3378) in NSW and 7.5% in Sweden (n=7622), with two thirds of these dispensed OCS (67% NSW, 69% Sweden), one quarter dispensed ≥5 SABA medications (25% NSW, 28% Sweden), while 24% of women in NSW and 14% in Sweden had an unplanned medical visit for asthma. Women could experience multiple types of exacerbation. The adjusted HRs (adjHR) for prenatal exacerbations and offspring asthma were 1.47 (95% CI 1.32 to 1.64) NSW and 1.30 (95% CI 1.24 to 1.36) Sweden (table 1). Paternal exacerbations also correlated with higher offspring asthma risks, adjHR 1.25 (95% CI 1.10 to 1.43) NSW and adjHR 1.18 (95% CI 1.12 to 1.24) Sweden. In the sibling analyses, associations for offspring asthma in Sweden moved towards the null, adjHR 1.09 (95% CI 0.97 to 1.23) (table 1). In NSW, the number of discordant pairs with complete confounder information was too small to use reliably (n=52). No differences were observed for sex when including interaction terms in models.

**Table 1:** Associations between prenatal asthma exacerbations and offspring asthma, HRs and 95% CIs

	NSW N=35 194			Sweden N=102 248		
	Unadjusted HR	Adjusted HR*	Adjusted HR†	Unadjusted HR	Adjusted HR*	Adjusted HR†
At least one exacerbation during pregnancy	1.49 (1.37 to 1.61)	1.52 (1.36 to 1.69)	1.47 (1.32 to 1.64)	1.42 (1.36 to 1.49)	1.38 (1.31 to 1.44)	1.30 (1.24 to 1.36)
Increasing number of exacerbations during pregnancy	1.20 (1.15 to 1.25)	1.21 (1.15 to 1.28)	1.19 (1.13 to 1.26)	1.35 (1.31 to 1.41)	1.32 (1.27 to 1.37)	1.26 (1.21 to 1.31)
Paternal control§‡	N=12 501			N=79 866		
	1.30 (1.14 to 1.48)	1.28 (1.12 to 1.46)	1.25 (1.10 to 1.43)	1.20 (1.15 to 1.26)	1.20 (1.14 to 1.26)	1.18 (1.12 to 1.24)
Sibling comparison‡	NA			N=1663 discordant pairs		
	NA	NA	NA	1.10 (0.98 to 1.23)	1.09 (0.97 to 1.23)	1.09 (0.97 to 1.23)

* BMI, SES, season of birth, smoking in pregnancy, maternal country of birth.

† Asthma severity.

‡ At least one exacerbation versus none.

§ BMI and smoking not available for paternal control.

BMI, body mass index; NA, not available; NSW, New South Wales; SES, socioeconomic status.

Among maternal asthma cohorts, prenatal ICS use was 28% in NSW and 32% in Sweden. The associations between prenatal ICS use (at least one dispensing compared with none) and offspring asthma were adjHR 1.37 (95% CI 1.26 to 1.49) NSW and adjHR 1.23 (95% CI 1.19 to 1.27) Sweden, after adjusting for asthma severity and exacerbations (table 2). Positive associations were also observed for increasing number of ICS dispensings (adjHR 1.12, 95% CI 1.09 to 1.16 NSW, adjHR 1.08, 95% CI 1.07 to 1.09 Sweden) and for paternal ICS use and offspring asthma: adjHR 1.20 (95% CI 1.09 to 1.31) NSW and adjHR 1.15 (95% CI 1.11 to 1.19) Sweden. In the sibling analyses associations for offspring asthma moved to the null, adjHR 1.26 (95% CI 0.69 to 2.32) NSW, 0.99 (95% CI 0.91 to 1.08) Sweden (table 2). No differences were observed for sex when including interaction terms in models.

**Table 2:** Associations between prenatal ICS use and offspring asthma, HRs and 95% CIs

	NSW N=35 194			Sweden N=102 248		
	**Unadjusted HR**	Adjusted HR*	**Adjusted HR†**	**Unadjusted HR**	Adjusted HR*	**Adjusted HR†**
At least one ICS dispensing during pregnancy	1.48 (1.40 to 1.56)	1.53 (1.42 to 1.65)	1.37 (1.26 to 1.49)	1.38 (1.34 to 1.42)	1.39 (1.35 to 1.43)	1.23 (1.19 to 1.27)
Increasing number of ICS dispensings during pregnancy	1.15 (1.13 to 1.17)	1.17 (1.14 to 1.20)	1.12 (1.09 to 1.16)	1.14 (1.12 to 1.15)	1.14 (1.13 to 1.15)	1.08 (1.07 to 1.09)
Paternal control‡§	N=12 234			N=79 866		
	1.28 (1.18 to 1.39)	1.29 (1.19 to 1.41)	1.20 (1.09 to 1.31)	1.23 (1.19 to 1.27)	1.23 (1.19 to 1.27)	1.15 (1.11 to 1.19)
Sibling comparison‡	220 discordant pairs			1790 discordant pairs		
	0.94 (0.70 to 1.27)	1.01 (0.58 to 1.74)	1.26 (0.69 to 2.32)	1.00 (0.92 to 1.08)	1.00 (0.91 to 1.08)	0.99 (0.91 to 1.08)

* BMI, socioeconomic status, season of birth.

† Asthma severity, asthma exacerbations in pregnancy.

‡ At least one ICS dispensing versus none.

§ BMI not available for paternal control.

BMI, body mass index; ICS, inhaled corticosteroid; NSW, New South Wales.

Associations remained consistent when extending ICS exposure to 1 month before pregnancy (online supplemental Table S3). Sensitivity analyses for alternative definitions of asthma severity and restricting the asthma population yielded similar results to the main analysis (online supplemental Tables S4 and S5). Women without reported BMI data had slightly lower odds for all outcomes (online supplemental Table S6) than women with BMI data in unadjusted and adjusted analyses, which may suggest that the main findings are slightly overinflated.

## Discussion

This study which applied harmonised methods to data from two countries described maternal asthma (exacerbations and ICS use) before, during and after pregnancy and assessed the impact on child offspring asthma. The main findings were that both maternal exacerbations and ICS use during pregnancy were associated with offspring asthma, even after adjusting for asthma severity defined in two different ways. However, paternal analyses were also positive, although smaller in magnitude, suggesting that the maternal associations may be confounded in part by unmeasured familial factors such as genes or shared environment. Further support for this conclusion was provided by the sibling analyses which controlled for familial confounding and found no increased risk of offspring asthma between siblings exposed to maternal exacerbations and ICS use.

In the descriptive analyses, asthma exacerbation rates in both population cohorts declined during pregnancy, peaked immediately post partum, followed by a gradual increase. ICS dispensing rates on the other hand were mostly steady across pregnancy with a slight dip at the beginning and a sharp decline post partum which slowly returned to prepregnancy levels. The exacerbation spike at the beginning of pregnancy is unexpected and could suggest that OCS increases fertility,^33^ although the increase in NSW begins before conception and peaks during the first month seemingly ‘after’ conception. This may be attributable to the fact that conception is measured 2 weeks after the last menstrual period. The overall exacerbation rate decline observed in pregnancy could lead to the conclusion that exacerbations really do decrease in pregnancy. Alternatively, the observed decline in OCS dispensing may indicate a reluctance to prescribe and use OCS due to concerns for poor outcomes such as preterm birth.^34 35^ The accompanying increase in unplanned hospital visits (particularly in NSW) would suggest that there are still women having serious exacerbations and there may be others that are having more mild exacerbations but are not taking OCS nor visiting hospital services. The immediate jump in OCS prescriptions in the first month postpartum would support this theory if women were having exacerbations and went back to taking OCS after birth. The other possibility is that postpartum hormone changes trigger asthma exacerbations, but if this was the case we would likely see an increase in ICS dispensings at the same time when in fact we observe the opposite.

We are not aware of any other studies that have measured exacerbations over the course of pregnancy but several small but well-characterised studies have shown that up to 40% of women with asthma have worsening symptoms over pregnancy.^1 2^ Studies of six cohorts also observed that OCS use decreased over pregnancy particularly in Italy and the Netherlands, however a sharp spike postpartum was not observed.^14 15^ This may be because the postpartum measurements were averaged over 3 months whereas our study measured per month.

ICS dispensing patterns were fairly similar to those observed in other cohorts with a slight decline in trimester one, returning to prepregnancy levels in trimester two.^14^,^16^ Our study with more recent data suggests that ICS adherence may be improving in pregnancy as the decline is smaller than that observed in a meta-analysis.^16^ However, the consistency between our results and previous results for a large downward spike in ICS dispensing immediately postpartum continues to be cause for alarm that women in this often very intense period may be more at risk of exacerbations and uncontrolled asthma. The fact that we observed a spike in OCS dispensing supports this. Therefore, we recommend that asthma management is preplanned for this vulnerable period such as preordering medications, arranging telehealth appointments etc.

The main findings assessing causal pathways support studies showing that prenatal uncontrolled and severe asthma are associated with offspring asthma and pneumonia compared with mild controlled asthma.^3 20^ However, the paternal control and sibling comparison analyses suggest that these associations are not causal but are explained by familial confounding at least in part. The source of this familial confounding is likely genetic given that asthma is a complex polygenic trait, therefore asthma exacerbation, an important clinical presentation of asthma, is likely to have a heritable component. We attempted to control for genetics with two definitions of asthma severity. However, it is likely that a genetic component remains, and indeed genetic propensity was identified as a possible source of confounding in the DAG (supplemental material). Another possible source of familial confounding is shared environmental triggers for mother and child (and father) such as pollution, mould, living conditions and respiratory infections.

Similarly to the exacerbation findings, our study found that prenatal ICS was associated with offspring asthma in mothers with asthma, even after adjusting for asthma exacerbations and severity. Again, the paternal control and sibling comparison studies suggest the associations may not be causal but rather are impacted by familial confounding, either genetics or environment. Our study stands in contrast to a clinical cohort from Australia that found that child asthma risk was reduced when mothers used ICS in pregnancy compared with those who did not,^22^ but is supported by a recent study of infants that found no association between prenatal ICS use and wheeze and bronchiolitis at 12 months of age.^36^ Therefore, our study does not support the hypothesis that ICS use in pregnancy may reduce offspring asthma risk. Nor does this study support a negative association, thus confirming the safety of ICS in pregnancy in regard to child asthma, and given the benefits of ICS during pregnancy, current guidelines to maintain ICS use remain appropriate.^18 37^ Further, ICS use is now recommended for all people with asthma.^38^ This study provides no reason to change this recommendation. Particularly because the rate of exacerbations during pregnancy and postpartum is so high, and optimal ICS use is likely to reduce the risk of exacerbations which have been shown to be associated with infant wheeze.^36^

The central strength of this study is the ability to use two population-wide cohorts from two geographically and ethnically diverse areas of the world with replicable results. Using register-based information meant we had little missing data, our results are generalisable, and we had the information required for covariate adjustment as well as for paternal and sibling analysis. Second, we were able to use an established validated algorithm to detect asthma in children and mothers.^29^ The main limitation was that due to reliance on administrative rather than clinical data we could not capture all exacerbations, only those that required extra medication use (OCS or ≥5 SABA) or a hospital visit. This means that there will be some misclassification of the unexposed group which could mean that any associations are underestimated, that is, closer to the null. Another limitation common to register-based studies is that we only have dispensing data and assume use of the medication. It is possible that women do not use their medication or are still using prescriptions dispensed earlier. The sensitivity analysis extended to dispensings 1 month prior to pregnancy did not show any differences, however, it is possible that any positive observed associations are biased towards the null due to lower-than-expected use. Finally, it should be acknowledged that although sibling designs reduce familial confounding they can potentially amplify non-shared confounding and random measurement error more than unpaired associations both of which can lead to attenuated associations.^39^ We attempted to reduce these sources of bias by adjusting for a number of non-shared confounders and using a validated asthma algorithm for the outcome and objective register-based data for the outcome, however, some bias may remain.

In two separate large populations, maternal exacerbations and ICS use in pregnancy were associated with increased childhood asthma risk. However, paternal and sibling analyses suggest that these associations may be confounded by familial factors, and therefore causal evidence remains inconclusive. While modifying ICS use or exacerbations in pregnancy is unlikely to reduce child asthma, monitoring of maternal asthma remains a priority to reduce exacerbation risk throughout pregnancy and post partum.

## Supplementary material

10.1136/bmjresp-2025-004010 online supplemental file 1

## Data Availability

Data are available on reasonable request.

## References

[1] Grosso A, Locatelli F, Gini E (2018). The course of asthma during pregnancy in a recent, multicase-control study on respiratory health. Allergy Asthma Clin Immunol.

[2] Stevens DR, Perkins N, Chen Z (2022). Determining the Clinical Course of Asthma in Pregnancy. J Allergy Clin Immunol Pract.

[3] Abdullah K, Zhu J, Gershon A (2020). Effect of asthma exacerbation during pregnancy in women with asthma: a population-based cohort study. Eur Respir J.

[4] Blais L, Kettani F-Z, Forget A (2015). Asthma exacerbations during the first trimester of pregnancy and congenital malformations: revisiting the association in a large representative cohort. Thorax.

[5] Kim S, Kim J, Park SY (2015). Effect of pregnancy in asthma on health care use and perinatal outcomes. J Allergy Clin Immunol.

[6] Bokern MP, Robijn AL, Jensen ME (2021). Factors Associated with Asthma Exacerbations During Pregnancy. J Allergy Clin Immunol Pract.

[7] Brew BK, Gibberd A, Marks GB (2023). Maternal asthma in Australian indigenous women and perinatal outcomes: A whole population-linked study. Int J Gynaecol Obstet.

[8] Murphy VE, Jensen ME, Gibson PG (2017). Asthma during Pregnancy: Exacerbations, Management, and Health Outcomes for Mother and Infant. Semin Respir Crit Care Med.

[9] Robijn AL, Brew BK, Jensen ME (2020). Effect of maternal asthma exacerbations on perinatal outcomes: a population-based study. ERJ Open Res.

[10] Rejnö G, Lundholm C, Gong T (2014). Asthma during pregnancy in a population-based study--pregnancy complications and adverse perinatal outcomes. PLoS One.

[11] Rejnö G, Lundholm C, Larsson K (2018). Adverse Pregnancy Outcomes in Asthmatic Women: A Population-Based Family Design Study. J Allergy Clin Immunol Pract.

[12] Namazy JA, Murphy VE, Powell H (2013). Effects of asthma severity, exacerbations and oral corticosteroids on perinatal outcomes. Eur Respir J.

[13] Chambers CD, Krishnan JA, Alba L (2021). The safety of asthma medications during pregnancy and lactation: Clinical management and research priorities. J Allergy Clin Immunol.

[14] Enriquez R, Wu P, Griffin MR (2006). Cessation of asthma medication in early pregnancy. Am J Obstet Gynecol.

[15] Charlton RA, Pierini A, Klungsøyr K (2016). Asthma medication prescribing before, during and after pregnancy: a study in seven European regions. BMJ Open.

[16] Robijn AL, Jensen ME, McLaughlin K (2019). Inhaled corticosteroid use during pregnancy among women with asthma: A systematic review and meta-analysis. Clin Exp Allergy.

[17] Robijn AL, Jensen ME, Gibson PG (2019). Trends in asthma self-management skills and inhaled corticosteroid use during pregnancy and postpartum from 2004 to 2017. J Asthma.

[18] Global Initiative for Asthma (2023). Global strategy for asthma management and prevention. www.ginasthma.org.

[19] Martel M-J, Rey E, Beauchesne M-F (2009). Control and severity of asthma during pregnancy are associated with asthma incidence in offspring: two-stage case-control study. Eur Respir J.

[20] Liu X, Agerbo E, Schlünssen V (2018). Maternal asthma severity and control during pregnancy and risk of offspring asthma. J Allergy Clin Immunol.

[21] Roff AJ, Robinson JL, Hammond SJ (2025). Maternal asthma during pregnancy and risks of allergy and asthma in progeny: A systematic review and meta-analysis. BJOG.

[22] Morten M, Collison A, Murphy VE (2018). Managing Asthma in Pregnancy (MAP) trial: FENO levels and childhood asthma. J Allergy Clin Immunol.

[23] Brew BK, Gong T, Williams DM (2017). Using fathers as a negative control exposure to test the Developmental Origins of Health and Disease Hypothesis: A case study on maternal distress and offspring asthma using Swedish register data. Scand J Public Health.

[24] D’Onofrio BM, Class QA, Rickert ME (2016). Translational Epidemiologic Approaches to Understanding the Consequences of Early-Life Exposures. Behav Genet.

[25] Tran DT, Robijn AL, Varney B (2024). Data Resource Profile: The Early Life Course data platform for research on perinatal and early childhood exposures and outcomes in Australia. Int J Epidemiol.

[26] Ludvigsson JF, Almqvist C, Bonamy A-KE (2016). Registers of the Swedish total population and their use in medical research. Eur J Epidemiol.

[27] Cnattingius S, Källén K, Sandström A (2023). The Swedish medical birth register during five decades: documentation of the content and quality of the register. Eur J Epidemiol.

[28] Ludvigsson JF, Otterblad-Olausson P, Pettersson BU (2009). The Swedish personal identity number: possibilities and pitfalls in healthcare and medical research. Eur J Epidemiol.

[29] Örtqvist AK, Lundholm C, Wettermark B (2013). Validation of asthma and eczema in population-based Swedish drug and patient registers. Pharmacoepidemiol Drug Saf.

[30] Australian Bureau of Statistics (2016). Socio-economic indexes for areas Canberra: Australian Bureau of Statistics. https://www.abs.gov.au/websitedbs/censushome.nsf/home/seifa.

[31] Global (2014). Global strategy for asthma management and prevention. www.ginasthma.org.

[32] Textor J, van der Zander B, Gilthorpe MS (2016). Robust causal inference using directed acyclic graphs: the R package ‘dagitty’. Int J Epidemiol.

[33] Michael AE, Papageorghiou AT (2008). Potential significance of physiological and pharmacological glucocorticoids in early pregnancy. Hum Reprod Update.

[34] Middleton PG, Gade EJ, Aguilera C (2020). ERS/TSANZ Task Force Statement on the management of reproduction and pregnancy in women with airways diseases. Eur Respir J.

[35] Flenady V, Middleton P, Smith GC (2011). Stillbirths: the way forward in high-income countries. The Lancet.

[36] Brew BK, Gibson PG, Collison AM (2025). Infant respiratory outcomes following asthma management and exacerbations in pregnancy. ERJ Open Res.

[37] Bendien SA, de Kruif MD, Feitsma H (2024). Summary of the Dutch Multidisciplinary Practice Guideline on Asthma and Pregnancy. J Allergy Clin Immunol Pract.

[38] Global Initiative for Asthma (2025). Asthma management and prevention for adults, adolescents and children 6-11 years (2025). a summary guide for healthcare providers.

[39] Frisell T (2021). Invited Commentary: Sibling-Comparison Designs, Are They Worth the Effort?. Am J Epidemiol.

