# Supplementary material for: Maternal asthma activity and offspring asthma: a linked-data population study in Australia and Sweden
Source: BMJ Open Respir Res. 2026 Jun 28;13(1):e004010. doi: 10.1136/bmjresp-2025-004010 (PMC13331117; doi:10.1136/bmjresp-2025-004010)
Supplement: online supplemental file 1 [file bmjresp-13-1-s001.docx]

# Supplement

## The role of asthma exacerbations and inhaled corticosteroid use in pregnancy on child asthma risk: a population cohort study in Australia and Sweden

Bronwyn K Brew^1,2,3^, Vanessa E Murphy^3^, Cecilia Lundholm^2^, Helga Zoega^4^, Alys Havard^5,6^, Annelies L Robijn^6^, Tong Gong^2^, Awad I Smew^2,7^, Gustaf Rejno^2^, Georgina Chambers^1^, Catarina Almqvist^2,8^

### Supplementary methods:

**Codes and data sources for asthma medications and diagnoses**

Asthma medications were identified from the Pharmaceutical Benefit Schedule Australia (https://pbs.gov.au) and the Prescribed Drug Register Sweden with the World Health Organization Anatomical Therapeutic Classification (ATC) codes R03AC (selective β-2 adrenoreceptor agonists- SABA), R03BA (Inhaled Corticosteroids – ICS), R03AK (Inhaled corticosteroids combined with long-acting β-2 agonists – ICS LABA), R03DC (leukotriene receptor agonists- LRTA) or R03DX (monoclonal antibodies). Primary diagnoses for asthma (International Classification of Diseases Code v10 J45, J46) were identified from the NSW Admitted Patient Data Collection (APDC), NSW Emergency Department Data Collection (EDDC) and the National Patient Register Sweden (NPR). In addition, SnoMed codes were used from the EDDC collection to identify asthma. These are shown below:

| 370218001 |
| --- |
| 427679007 |
| 389145006 |
| 281239006 |
| 312453004 |
| 442025000 |
| 708090002 |
| 266361008 |
| 405944004 |
| 18197001 |
| 195967001 |
| 195949008 |
| 782520007 |

**Figure S1. Directed Acyclic Graph. Asthma exacerbation in pregnancy and offspring asthma.** Minimal adjustment set: asthma severity, BMI, ethnicity, SES, smoking in pregnancy, season of birth, (genes). Created with dagitty.net/dags.html. (ethnicity = maternal country of birth)


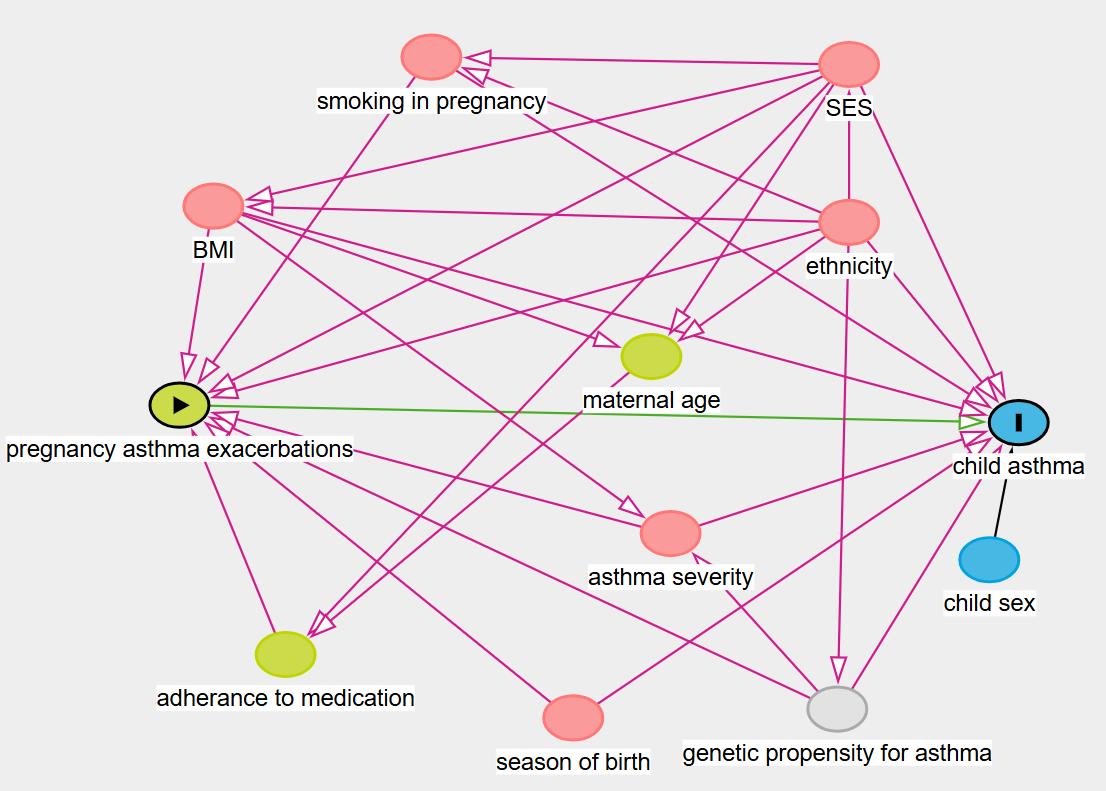


**Figure S2. Directed Acyclic Graph. ICS medication in pregnancy and offspring asthma.** Minimum adjustment set: asthma severity, asthma exacerbations in pregnancy, season of birth, BMI, SES. (created with dagitty.net/dags.html) . (ethnicity = maternal country of birth)


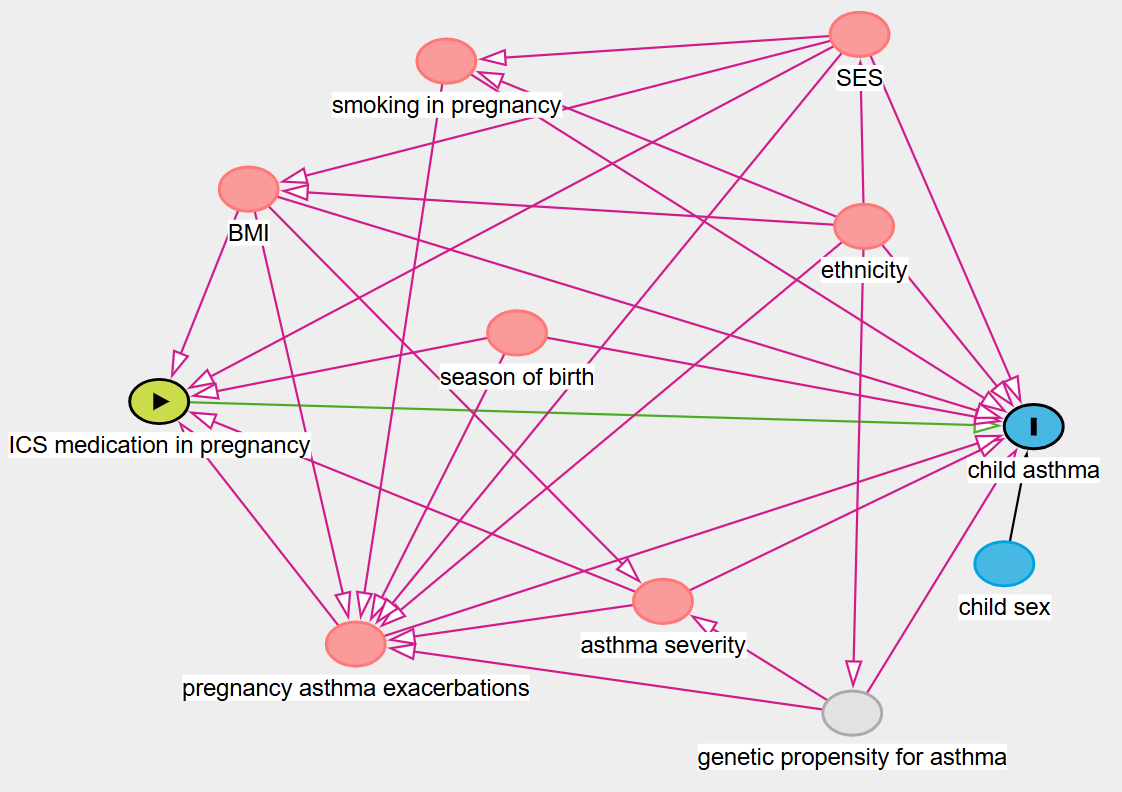


**Figure S3. Rate of ICS dispensings per month** in the asthma maternal and paternal cohorts from NSW (Oct 2014- Dec 2018) and Sweden (Jan 2008- Dec 2021), during pregnancy to 12 months post-birth.


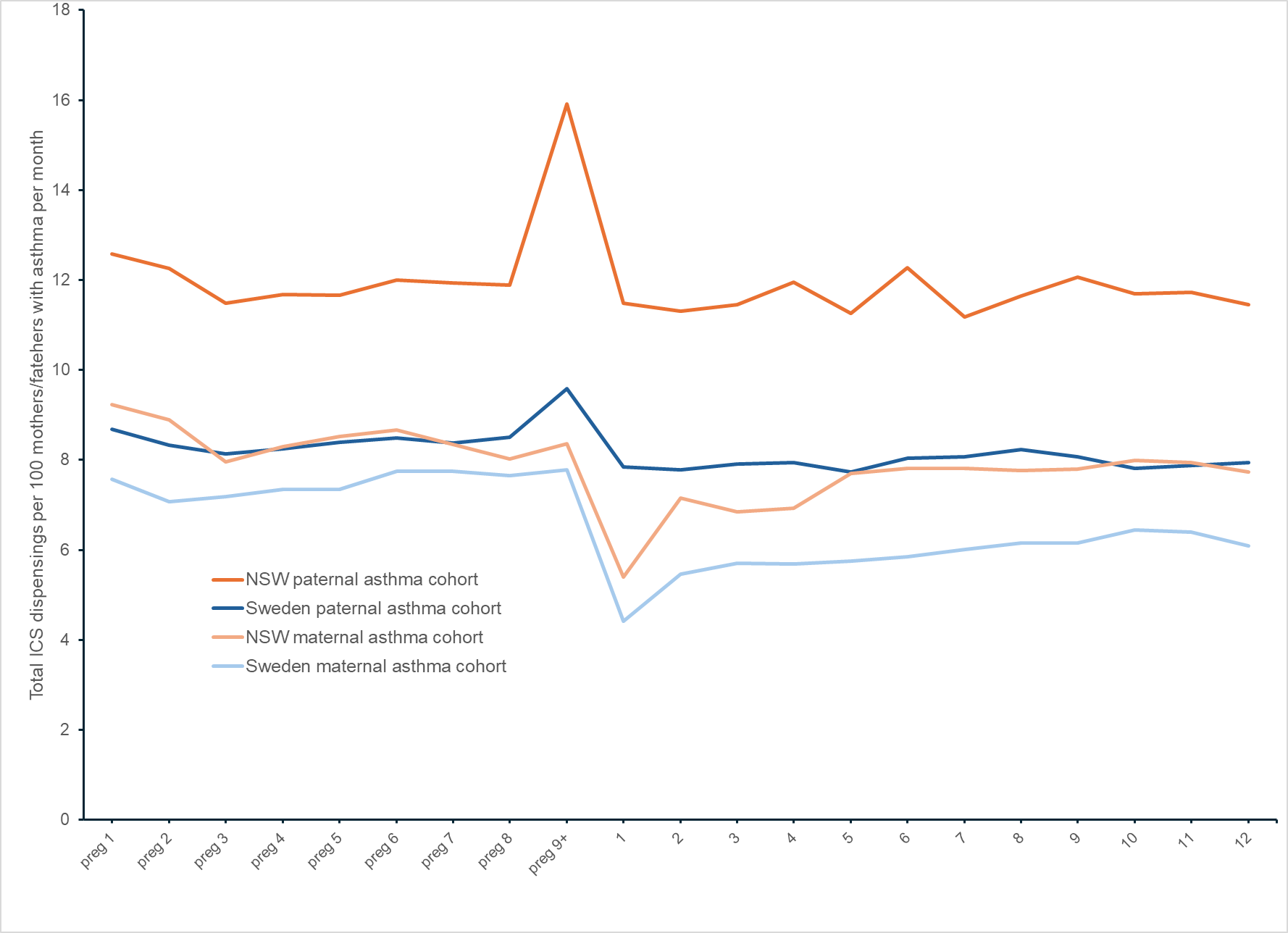


**Table S1. Characteristics of Population cohorts of pregnant women in NSW (n= 407 879) and Sweden (n=1 443 139).**

| **Characteristic** |  | **NSW** | **Sweden** |
| --- | --- | --- | --- |
| **Maternal Age (Mean, SD)** |  | 30.7 (5.4) | 30.5 (5.2) |
| **BMI, n (%)** | Underweight | 12 784 (4.6) | 33 729 (2.5) |
|  | Recommended | 149 217 (53.7) | 785 855 (57.8) |
|  | Overweight | 65 827 (23.7) | 350 844 (25.8) |
|  | Obese | 50 145 (18.0) | 189 373 (13.9) |
|  | Missing | 129 906 | 83 339 |
| **SES (SEIFA), n (%)** | Most disadvantaged quintile 1 | 88 619 (22.2) | - |
|  | 2 | 84 299 (21.1) | - |
|  | 3 | 79 564 (19.9) | - |
|  | 4 | 61 642 (15.4) | - |
|  | Least disadvantaged quintile 5 | 85 925 (21.5) | - |
|  | Missing | 7830 |  |
| **SES (Education), n (%)** | Compulsory education (≤ Year 9) | - | 153 314 (10.9) |
|  | Secondary education (Yr 10- 12) | - | 507 127 (36.0) |
|  | Any tertiary education | - | 749 464 (53.2) |
|  | Missing |  | 33 235 |
| **Maternal Country of birth, n (%)** | Nation of study site (Australia/Sweden) | 254 726 (62.6) | 1 067 710 (74.0) |
|  | Asia & Middle East | 100 060 (24.6) | 160 516 (11.1) |
|  | Europe (not Sweden) | 22 661 (5.6) | 92 131 (6.4) |
|  | Other | 29 725 (7.3) | 122 537 (8.5) |
|  | Missing | 707 | 246 |
| **Smoking in pregnancy, n (%)** |  | 26 170 (6.4) | 71 969 (5.2) |
| **Asthma, n (%)** |  | 35 194 (8.6) | 102 248 (7.1) |
| **Sex of baby (female), n (%)** |  | 198 071 (48.6) | 701 197 (48.6) |

SEIFA = socio-economic index for area

**Table S2. Characteristics of Maternal asthma cohorts (mothers and babies) in NSW and Sweden and exposure groups (prenatal ICS use, prenatal asthma exacerbations)**

|  |  | **NSW** | | | **Sweden** | | |
| --- | --- | --- | --- | --- | --- | --- | --- |
|  |  | **Asthma cohort**  N= 35 194 | **≥1 asthma exacerbation**  N= 3378 | **≥1 ICS dispensing**  N= 9932 | **Asthma cohort**  N= 102 248 | **≥1 asthma exacerbation**  N= 7622 | **≥1 ICS dispensing**  N= 33 021 |
| **Maternal Age (Mean, SD)** |  | 30.3 (5.9) | 30.4 (1.9) | 31.3 (5.7) | 30.8 (5.3) | 31.3 (5.6) | 31.5 (5.2) |
| **Maternal BMI, n (%)** | Underweight | 839 (3.5) | 63 (2.7) | 175 (2.6) | 1755 (1.8) | 130 (1.8) | 577 (1.9) |
|  | Recommended | 10 175 (42.3) | 850 (36.6) | 2766 (41.5) | 48 462 (50.4) | 3191 (44.7) | 15 496 (49.6) |
|  | Overweight | 6200 (25.8) | 581 (25.0) | 1744 (26.1) | 94 478 (28.1) | 2051 (28.8) | 8766 (28.1) |
|  | Obese | 6815 (28.4) | 829 (35.7) | 1988 (29.8) | 19 025 (19.8) | 1760 (24.7) | 6385 (20.5) |
|  | Missing | 11 165 | 1055 | 3259 | 6015 | 490 | 1797 |
| **SES (SEIFA), n (%)** | Most Disadvantaged 1 | 8880 (25.4) | 1040 (30.9) | 2220 (22.5) | - | - | - |
|  | 2 | 8381 (24) | 918 (27.3) | 2284 (23.1) | - | - | - |
|  | 3 | 6480 (18.6) | 588 (17.5) | 1855 (18.8) | - | - | - |
|  | 4 | 4732 (13.6) | 378 (11.3) | 1364 (13.8) | - | - | - |
|  | Least disadvantaged 5 | 6445 (18.5) | 437 (13.0) | 2146 (21.7) | - | - | - |
|  | Missing | 276 | 17 | 63 |  | | |
| **SES (Education), n (%)** | Compulsory education (≤ Year 9) | - | - | - | 9697 (9.6) | 959 (12.7) | 2562 (7.8) |
|  | Secondary education Yr 10- 12 | - | - | - | 38 011 (37.4) | 2984 (39.4) | 11 036 (33.6) |
|  | Any tertiary education | - | - | - | 53809 (53.0) | 3626 (47.9) | 19 236 (58.6) |
|  | Missing |  |  |  | 731 | 53 | 187 |
| **Maternal country of birth, n (%)** | Nation of study site (Australia/Sweden) | 27 716 (78.9) | 2752 (81.5) | 8062 (81.3) | 87 362 (85.4) | 6655 (87.3) | 29 021 (87.9) |
|  | Asia & Middle East | 4143 (11.8) | 350 (10.4) | 596 (6.0) | 6175 (6.0) | 430 (5.6) | 1639 (5.0) |
|  | Europe (not Sweden) | 1187 (3.4) | 82 (2.4) | 335 (3.4) | 4158 (4.1) | 256 (3.4) | 1105 (3.4) |
|  | Other | 2098 (5.9) | 193 (5.7) | 928 (9.3) | 4551 (4.5) | 281 (3.7) | 1256 (3.8) |
|  | Missing | 50 | 1 | 11 | 2 | 0 | 0 |
| **Smoking in pregnancy, n (%)** |  | 4494 (12.8) | 668 (19.8) | 1133 (11.4) | 6985 (7.2) | 816 (11.3) | 1833 (5.8) |
| **Sex of baby (female), n (%)** |  | 17 185 (48.8) | 1638 (48.5) | 4794 (48.3) | 49 914 (48.8) | 3703 (48.6) | 16 2 75 (49.3) |
| **Preterm birth (< 37 weeks), n (%)** |  | 3219 (9.2) | 372 (11.1) | 847 (8.5) | 6790 (6.6) | 598 (7.9) | 2088 (6.3) |
| **Low birth weight (<2500g), n (%)** |  | 2794 (7.9) | 319 (9.4) | 783 (7.9) | 4977 (4.9) | 462 (6.1) | 1656 (5.0) |
| **Asthma severity, n (%)** | SABA only | 11 115 (31.6) | 695 (20.6) | 734 (7.4) | 35 307 (34.5) | 1643 (21.6) | 2804 (8.5) |
|  | ICS monotherapy | 3478 (9.9) | 266 (7.9) | 926 (9.3) | 33 579 (32.8) | 2182 (28.6) | 11 912 (36.1) |
|  | ICS combinations (LABA or LRTA) | 19 958 (56.7) | 2346 (69.5) | 8214 (82.7) | 32 522 (31.8) | 3719 (48.8) | 18 180 (55.1) |
|  | No medication | 643 (1.8) | 71 (2.1) | 58 (0.6) | 840 (0.8) | 78 (1.0) | 125 (0.4) |

SEIFA = socio-economic index for area ^30^

**Table S3. Sensitivity analysis extending exposure period for an ICS dispensing to one month prior to conception. Hazard Ratios and 95%CI .**

|  | **NSW**  **N= 35 144** | | **Sweden**  **N= 102 175** | |
| --- | --- | --- | --- | --- |
|  | **Unadjusted HR (95%CI)** | **Adjusted HR^a^** | **Unadjusted HR (95%CI)** | **Adjusted HR^a^** |
| **At least one ICS dispensing during pregnancy** | 1.46 (1.39, 1.54) | 1.33 (1.22, 1.44) | 1.38 (1.35, 1.42) | 1.23 (1.19, 1.27) |

a = body mass index, socioeconomic status, season of birth, asthma severity, asthma exacerbations in pregnancy

**Table S4. Sensitivity analysis for asthma severity. ICS use in pregnancy and asthma in children in NSW and Sweden. Subgroup of women who had two or more ICS dispenses in the 27 months prior to conception. Severity measured as pre-pregnancy defined daily dose of ICS. Hazard ratios and 95%CI**

|  | **NSW**  **N= 13 620** | | | **Sweden**  **N= 42 363** | | |
| --- | --- | --- | --- | --- | --- | --- |
|  | **Unadjusted HR** | **Adjusted HR^a^** | **Adjusted HR^b^** | **Unadjusted HR** | **Adjusted HR ^a^** | **Adjusted ^b^** |
| **At least one ICS dispensing during pregnancy** | 1.21 (1.12, 1.31) | 1.28 (1.15, 1.42) | 1.21 (1.09, 1.36) | 1.12 (1.08, 1.17) | 1.13 (1.08, 1.17) | 1.10 (1.06, 1.14) |
|  | **N= 5612** | | | **N= 34 381** | | |
| **Paternal control** | 1.13 (1.00, 1.28) | 1.14 (1.01, 1.29)* | 1.12 (0.99, 1.27)* | 1.08 (1.03, 1.13) | 1.08 (1.03, 1.14) | 1.07 (1.02, 1.12) |
|  |  | | | **N= 478** | | |
| **Sibling comparison** | NA | NA | NA | 0.90 (0.80, 1.00) | 0.90 (0.80, 1.01) | 0.89 (0.79, 1.01) |

* BMI not available for other parents. a = body mass index, socioeconomic status, season of birth. b = body mass index, socioeconomic status, season of birth, asthma severity, asthma exacerbations in pregnancy

**Table S5. Sensitivity analysis for asthma severity. Asthma exacerbation in pregnancy and asthma in children, in NSW and Sweden. Subgroup of women who had two or more ICS dispenses in the 27 months prior to conception. Severity measured as pre-pregnancy defined daily dose of ICS. Hazard ratios and 95%CI**

|  | **NSW**  **N= 13 631** | | | **Sweden**  **N= 42 363** | | |
| --- | --- | --- | --- | --- | --- | --- |
|  | **Unadjusted HR** | **Adjusted HR^a^** | **Adjusted HR^b^** | **Unadjusted HR** | **Adjusted HR ^a^** | **Adjusted ^b^** |
| **At least one exacerbation during pregnancy** | 1.41 (1.28, 1.56) | 1.47 (1.28, 1.68) | 1.47 (1.28, 1.68) | 1.32 (1.25, 1.39) | 1.27 (1.20, 1.35) | 1.27 (1.19, 1.34) |
|  | **N= 5612** | | | **N= 34 381** | | |
| **Paternal control** | 1.18 (1.00, 1.38) | 1.14 (0.97, 1.34) | 1.14 (0.97, 1.35) | 1.13 (1.06, 1.20) | 1.12 (1.05, 1.20) | 1.13 (1.06, 1.21) |
|  |  | | | **N= 908** | | |
| **Sibling comparison** | NA | NA | NA | 1.07 (0.92, 1.23) | 1.06 (0.90, 1.24) | 1.06 (0.90, 1.24) |

* BMI not available for other parents. a = body mass index, socioeconomic status, season of birth. b = body mass index, socioeconomic status, season of birth, asthma severity, asthma exacerbations in pregnancy

**Table S6. Sensitivity analysis for missing BMI data. NSW maternal analyses with and without women who have data on BMI status.**

|  | Unadjusted HR  Women with BMI data  N= 23 998 | Unadjusted HR  Women without BMI data  N= 11 146 | Adjusted HR*  Women with BMI data  N= 23 702 | Adjusted HR*  Women without BMI data  N= 11 120 |
| --- | --- | --- | --- | --- |
| At least one exacerbation during pregnancy | 1.54 (1.38, 1.71) | 1.43 (1.26, 1.61) | 1.53 (1.37, 1.71) | 1.43 (1.26, 1.62) |
| Increasing number of exacerbations during pregnancy | 1.22 (1.16, 1.29) | 1.18 (1.11, 1.26) | 1.22 (1.15, 1.29) | 1.18 (1.11, 1.26) |
| At least one ICS dispensing during pregnancy | 1.52 (1.41, 1.63) | 1.42 (1.31, 1.55) | 1.54 (1.42, 1.66) | 1.44 (1.33, 1.57) |
| Increasing number of ICS dispensings during pregnancy | 1.17 (1.14, 1.20) | 1.12 (1.09. 1.16) | 1.17 (1.14, 1.21) | 1.13 (1.09, 1.16) |

* Exacerbation analyses adjusted for: SES, season of birth, smoking in pregnancy, maternal country of birth (without BMI). ICS use analyses adjusted for: SES, season of birth (without BMI).
